# Supplementary material for: Transcriptional profiling of mESC-derived tendon and fibrocartilage cell fate switch
Source: Nat Commun. 2021 Jul 9;12:4208. doi: 10.1038/s41467-021-24535-5 (PMC8270956; doi:10.1038/s41467-021-24535-5)
Supplement: Supplementary file 1 — Supplementary Information [file 41467_2021_24535_MOESM1_ESM.pdf]

Supplementary Figure 1

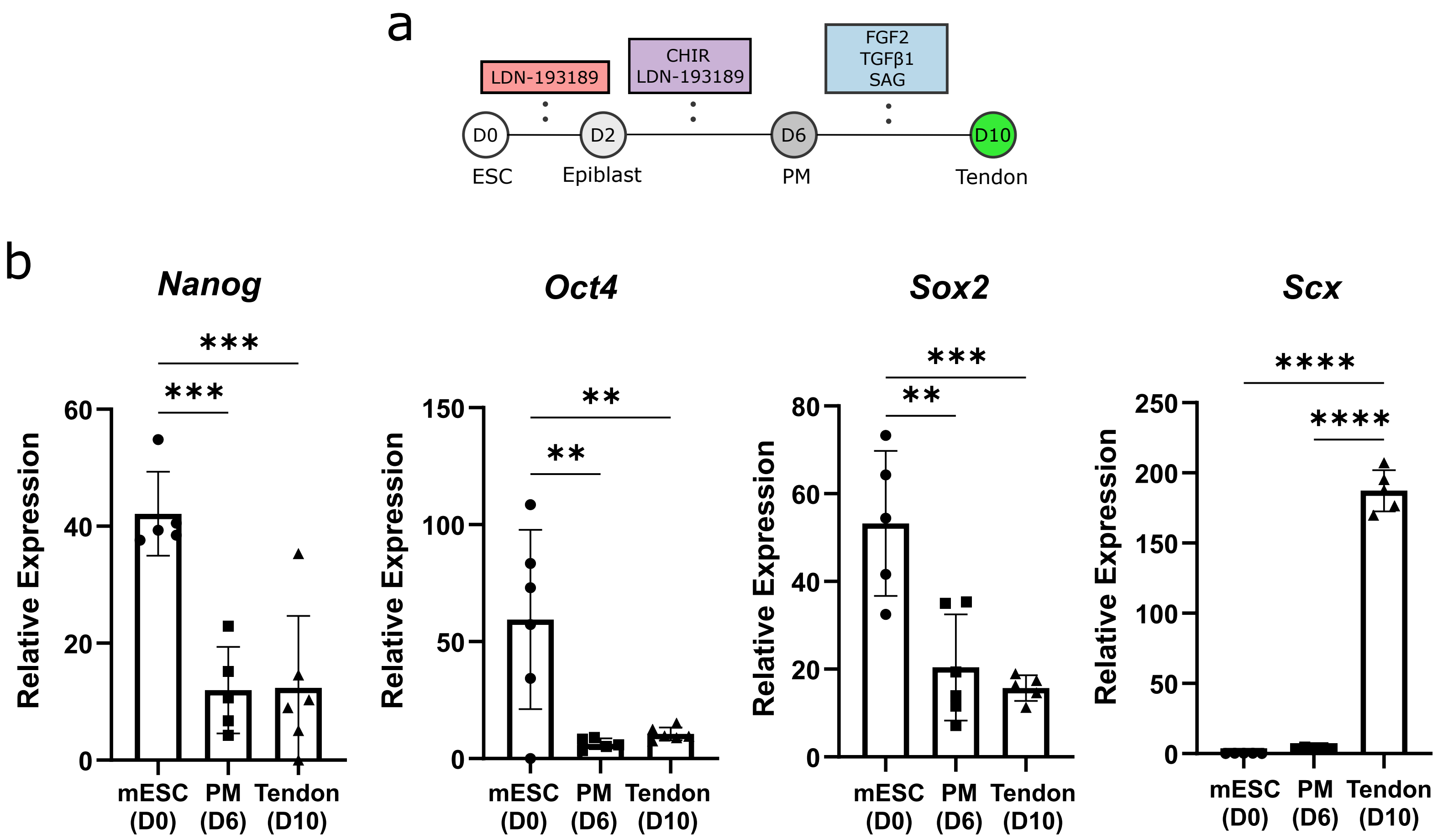

Supplementary Figure 1: Loss of pluripotent markers with PM and tenogenic differentiation. (A) Schematic of experimental design. (B) qPCR quantification of pluripotent markers (Nanog, Oct4, Sox2) and tendon marker (Scx) (n=6 independent samples, one way ANOVA with Tukey's posthoc tests). Data shown as mean+/-SD. **p<0.01 ***p<0.001 ****p<0.0001. Source data provided in source data file.

Supplementary Figure 2

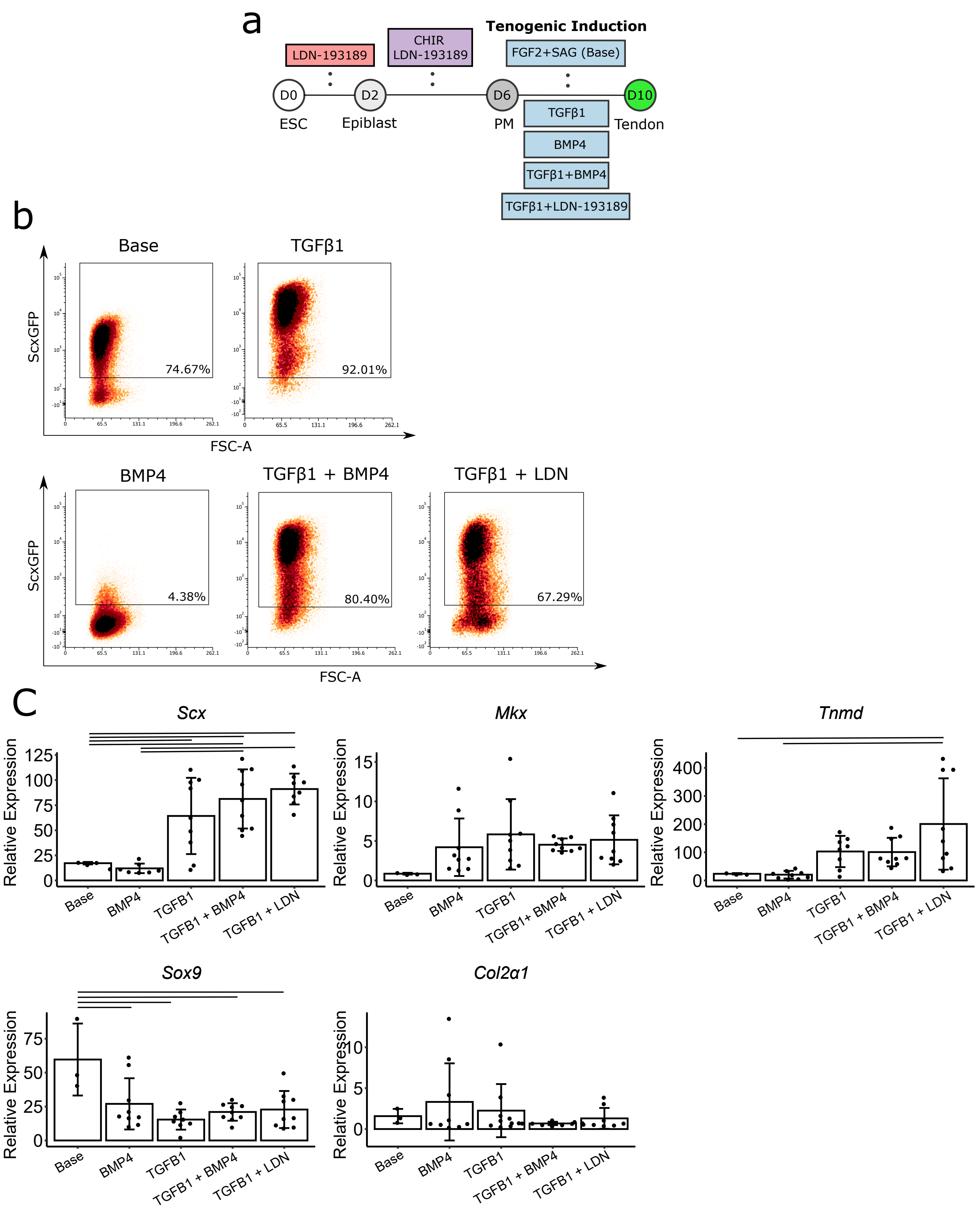

Supplementary Figure 2: TGFβ signaling modifies the anti-tenogenic effects of BMP4. (A) Schematic of BMP and TGFβ media conditions. Top row indicates common media components. Bottom row indicates media component variables. (B) Flow cytometry for ScxGFP (C) qPCR quantification of tendon (Scx, Mkx, Tnmd) and cartilage markers (Sox9, Col2a1) (n=3 independent samples for base condition, n=9 independent samples for all other conditions, one way ANOVA with Tukey's posthoc tests). Data shown as mean+/-SD. Bars indicate $p<0.05$. Source data provided in source data file.

Supplementary Figure 3

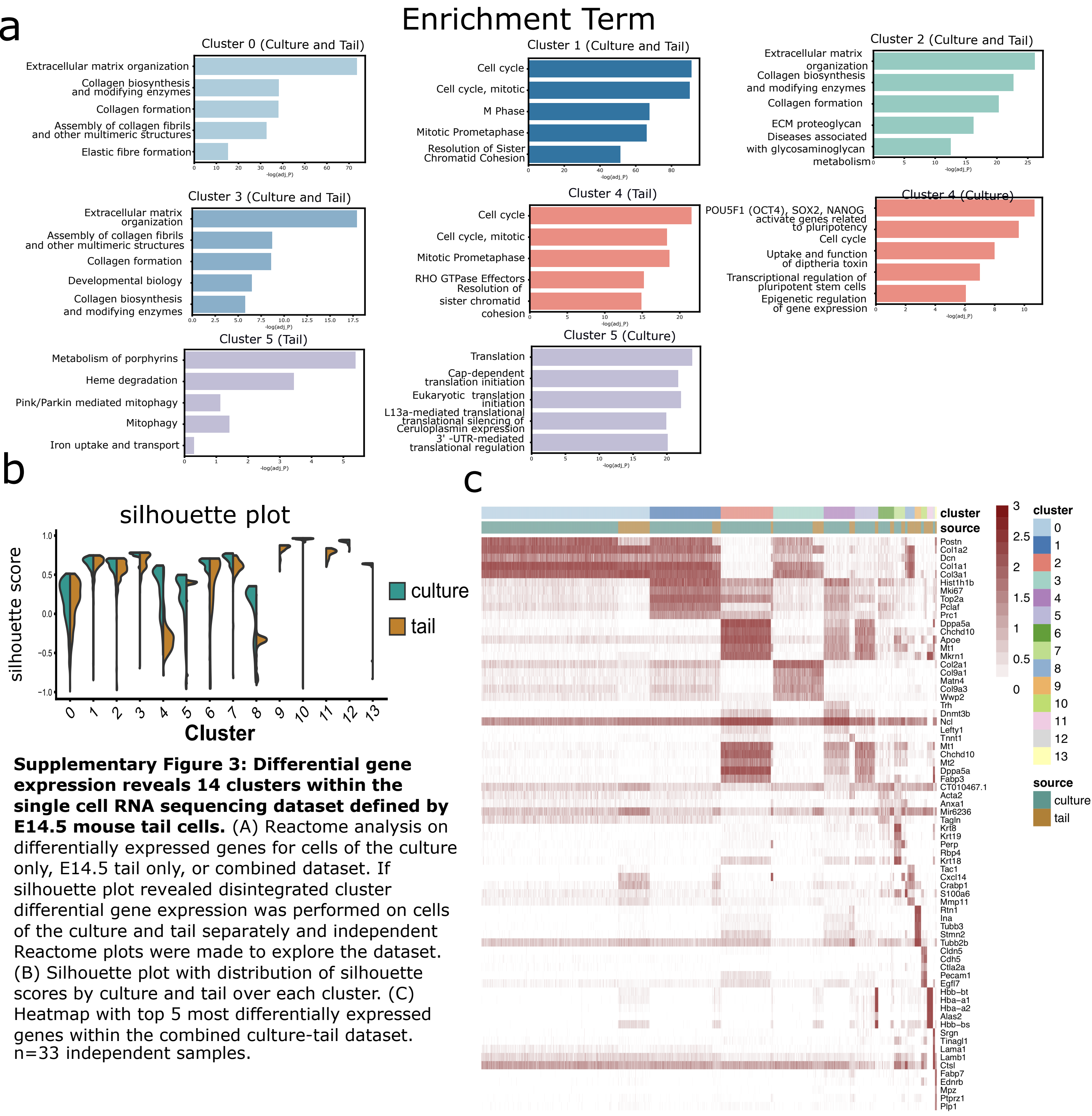

Supplementary Figure 3: Differential gene expression reveals 14 clusters within the single cell RNA sequencing dataset defined by E14.5 mouse tail cells. (A) Reactome analysis on differentially expressed genes for cells of the culture only, E14.5 tail only, or combined dataset. If silhouette plot revealed disintegrated cluster differential gene expression was performed on cells of the culture and tail separately and independent Reactome plots were made to explore the dataset. (B) Silhouette plot with distribution of silhouette scores by culture and tail over each cluster. (C) Heatmap with top 5 most differentially expressed genes within the combined culture-tail dataset. n=33 independent samples.

Supplementary Figure 4

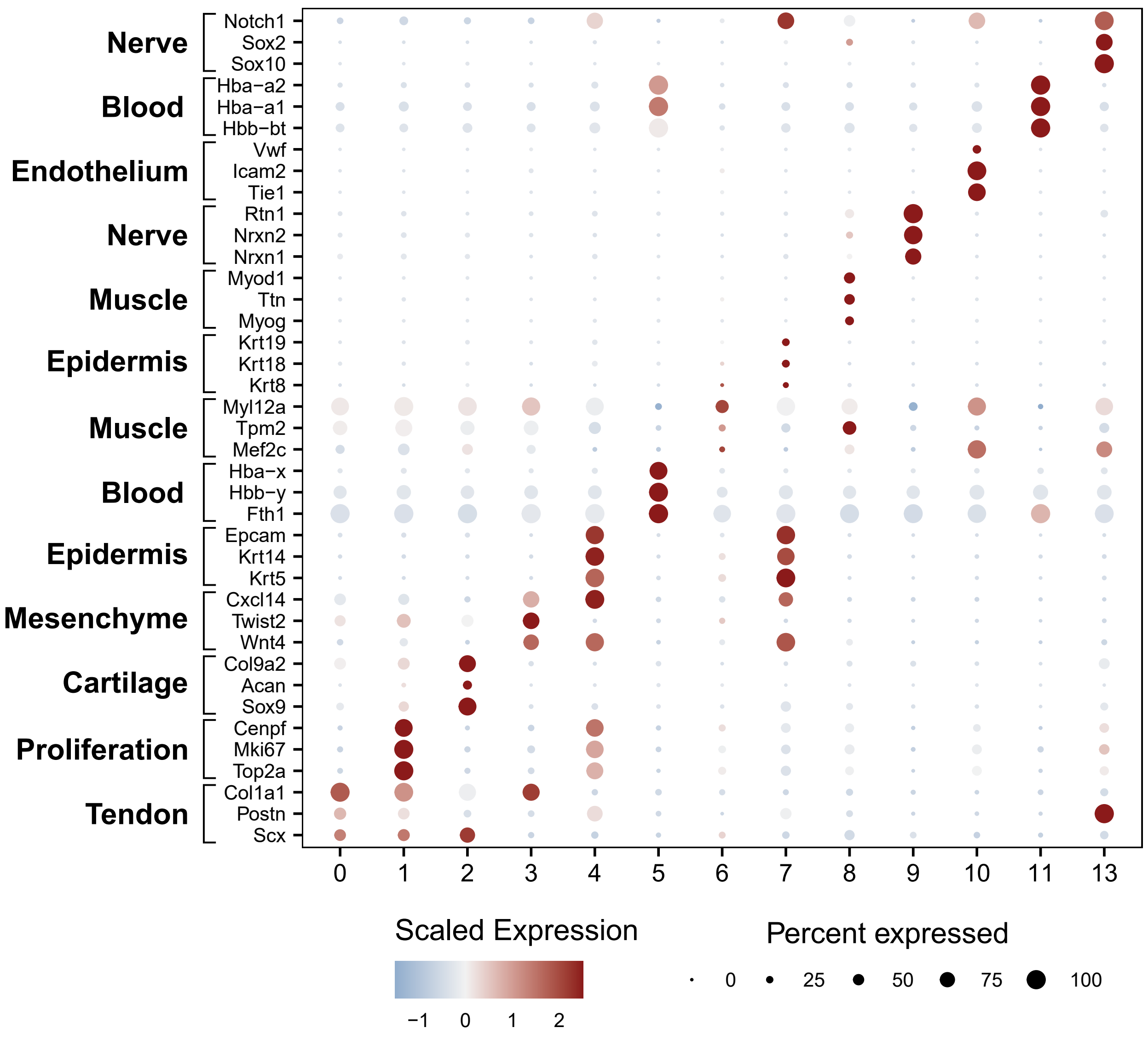

Supplementary Figure 4: Dot plot analysis based on the scRNASeq E14.5 tail reference dataset shows cluster identities. Cell phenotypes identified include tendon, proliferating cells, cartilage, mesenchyme, epidermis, blood, muscle, endothelium, and nerve cells.

Supplementary Figure 5

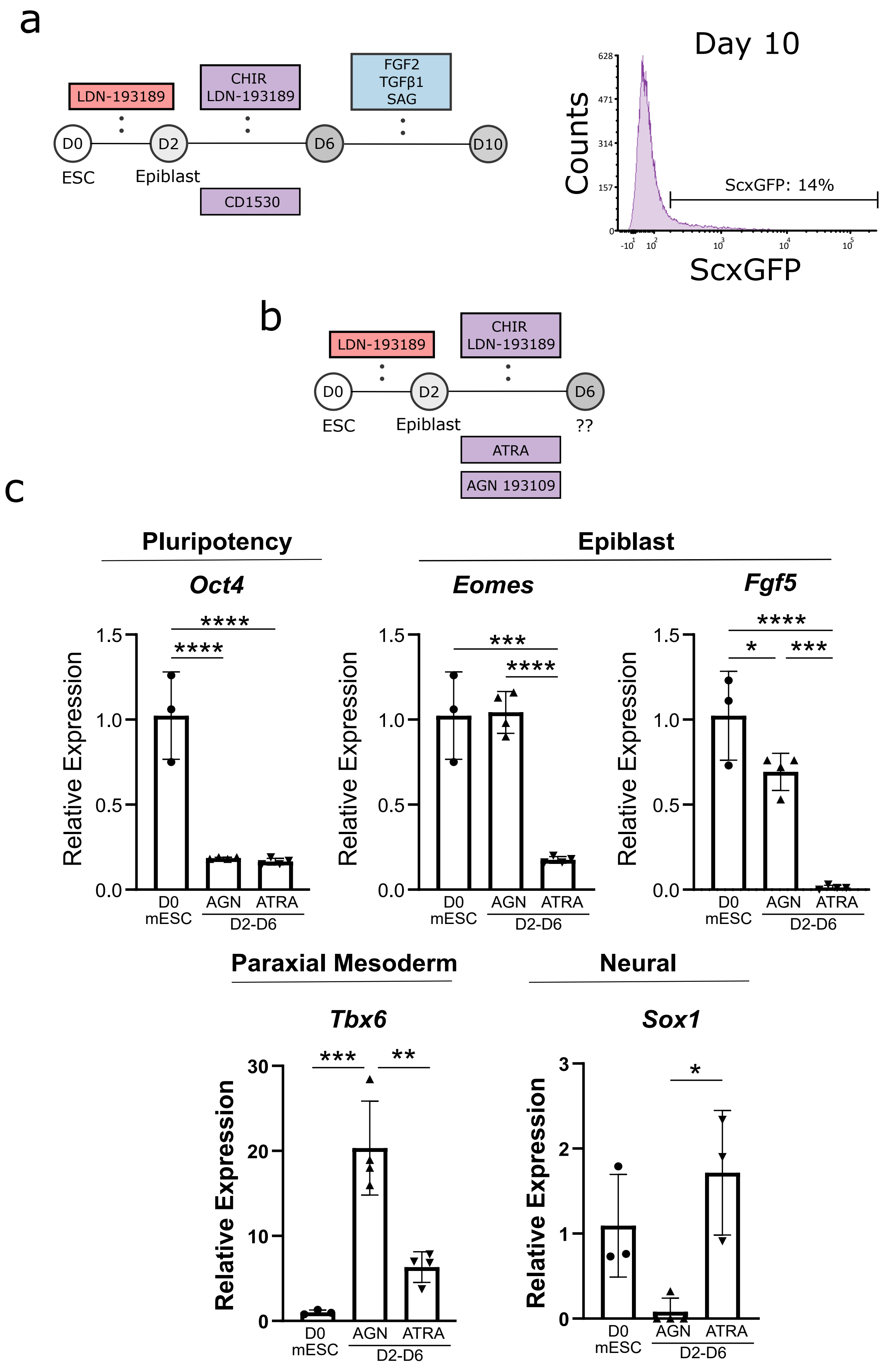

Supplementary Figure 5: Activation of retinoic acid signaling during D2-D6 results in loss of paraxial mesoderm induction and loss of tenogenic competence. (a) Schematic of experimental design and ScxGFP quantification at D10 with CD1530 treatment (D2-D6). (b) Schematic of experimental design of ATRA vs AGN treatment (D2-D6). (c) qPCR quantification of pluripotent (Oct4), epiblast (Eomes, Fgf5), paraxial mesoderm (Tbx6), and neural (Sox1) markers. (n=4 independent samples, one way ANOVA with Tukey's posthoc tests). Data shown as mean+/-SD. *p<0.05 **p<0.01 ***p<0.001 ****p<0.0001. Source data provided in source data file.

Supplementary Figure 6

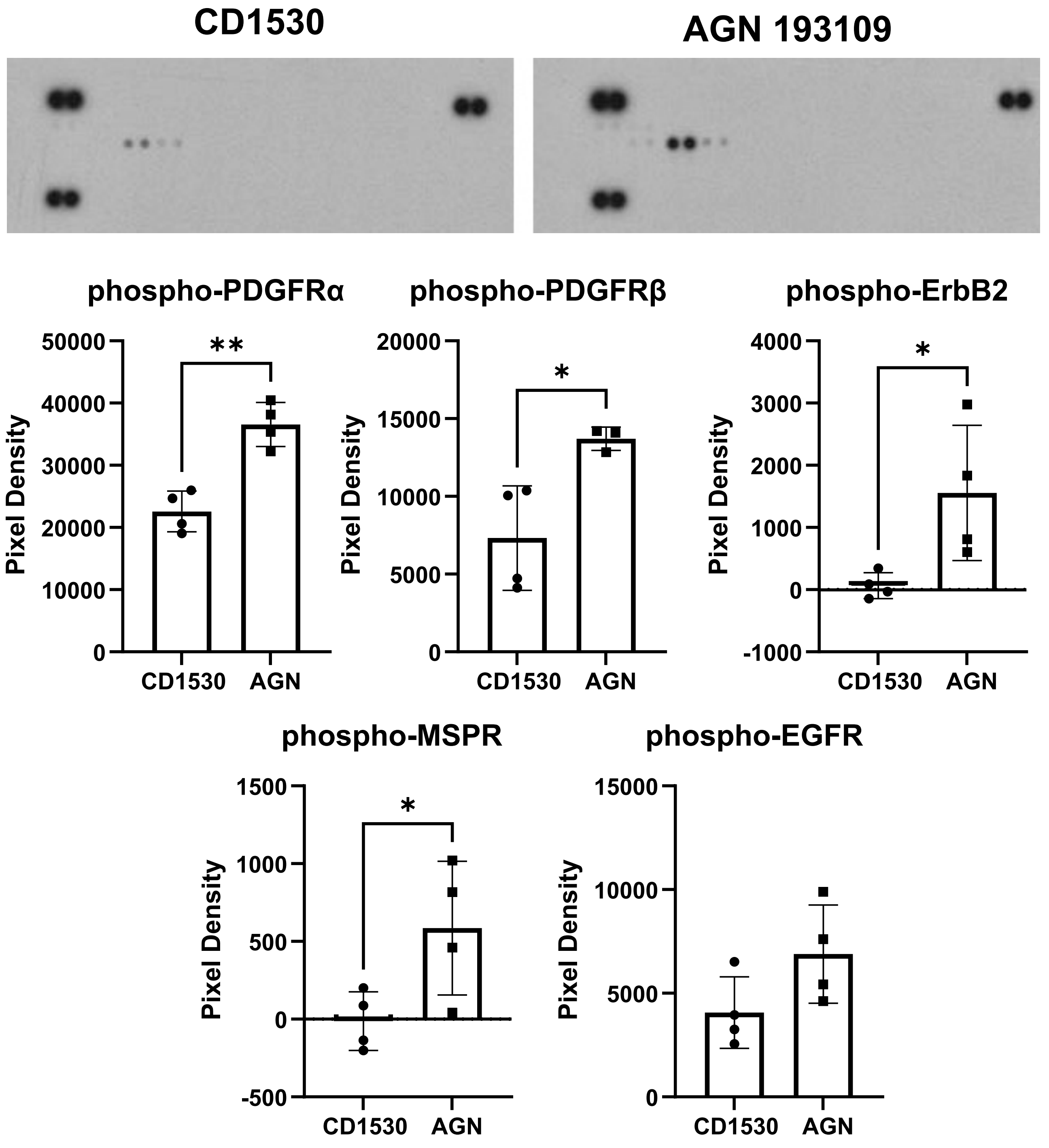

.

Supplementary Figure 6: Proteomic analysis of phosphorylated receptor tyrosine kinases. Representative blots and quantification of phosphorylated PDGFRa, PDGFRβ, ErbB2, MSP-R, and EGF-R (n=4 independent samples, unpaired two-sided Student's t-tests). Data shown as mean+/-SD. * $p<0.05$ ** $p<0.01$. Source data provided in source data file.

Supplementary Figure 7

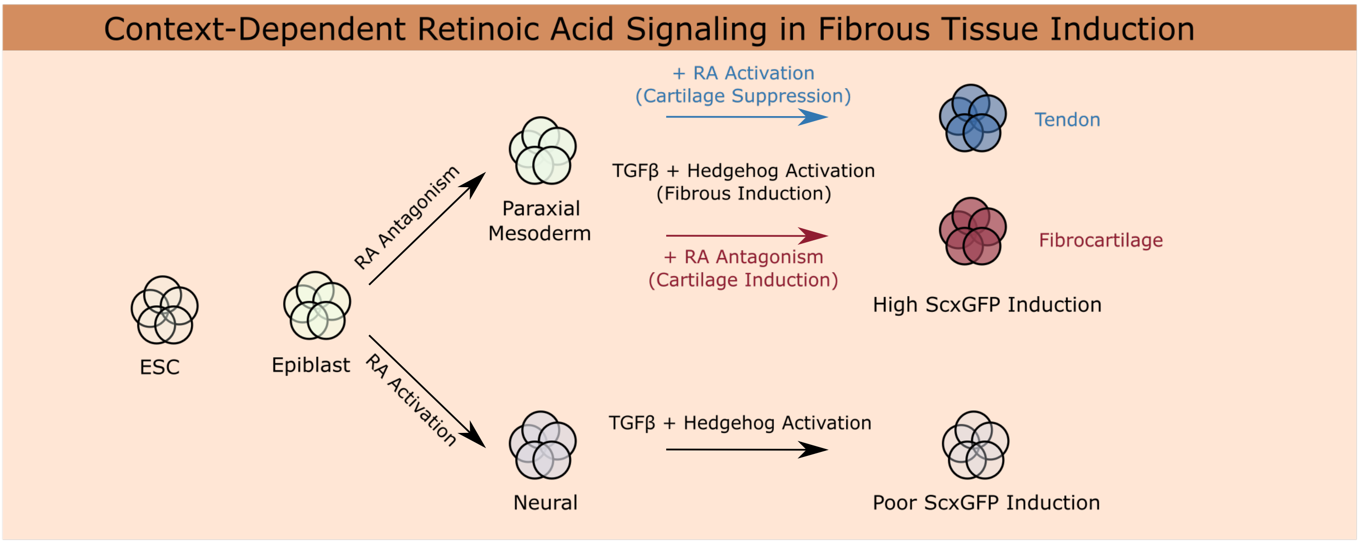

Supplementary Figure 7: Schematic overview of context-dependent retinoic acid signaling. The activity of retinoic acid activation or antagonism depends on cell competency as well as contextualizing signals from TGFβ and hedgehog activation.

Supplementary Figure 8

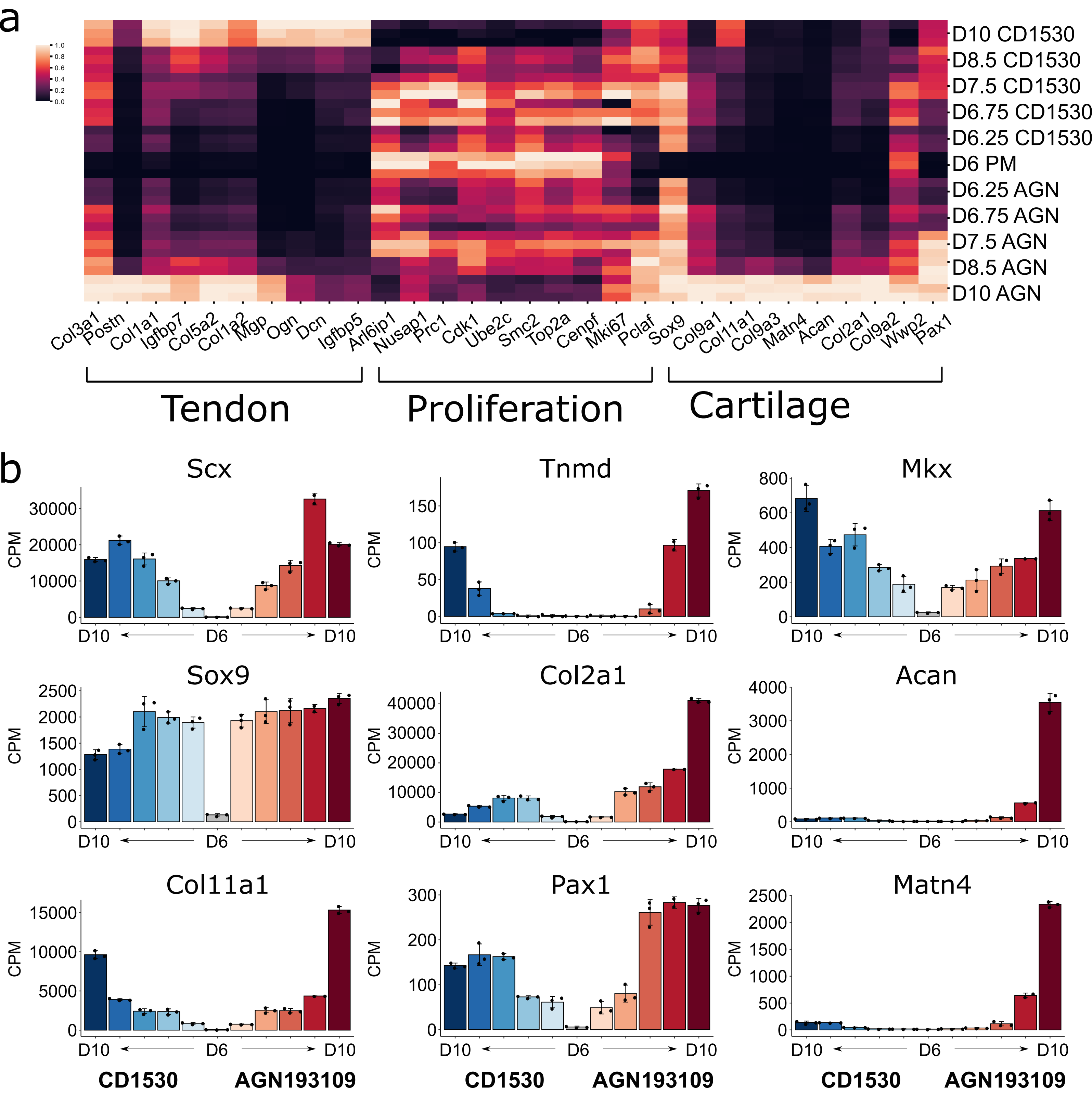

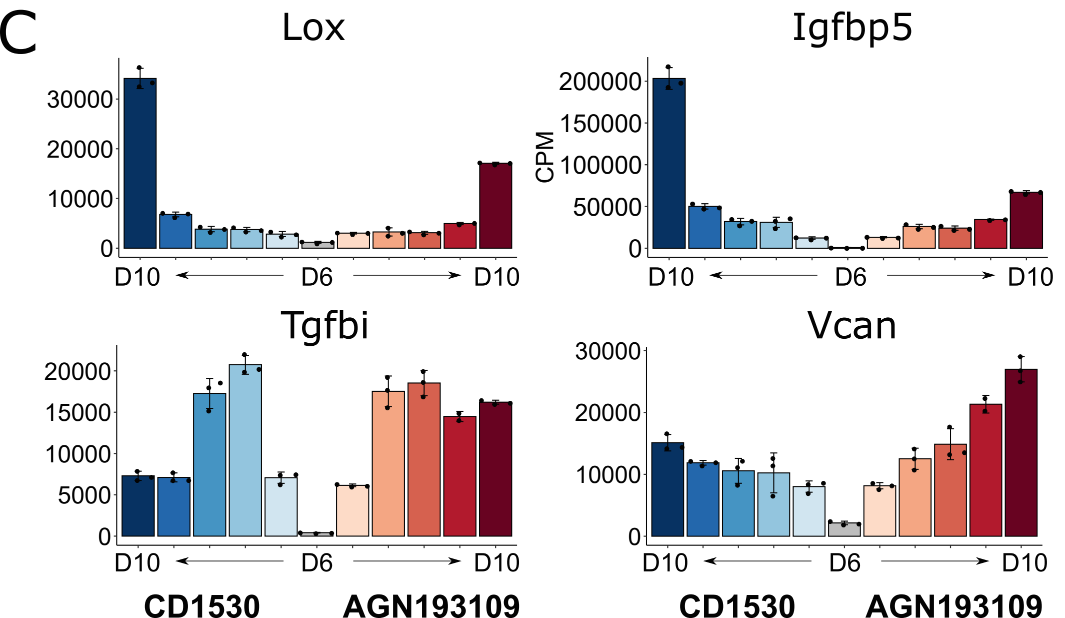

Supplementary Figure 8: Transcriptional profiling by RNA sequencing confirms acquisition of tendon and fibrocartilage specificity with retinoic acid manipulation. (A) Cluster 0 (tendon), cluster 1 (proliferating tendon), cluster 2 (fibrocartilage) identities defined by scRNA-seq of the E14.5 tail projected onto the RNA-seq dataset from CD1530 and AGN 193109 treated cells. (B) Tendon and cartilage markers identified from scRNA-seq signatures confirmed by RNA-seq trajectories. n=3 independent samples. (C) Genes identified from RNA-seq that strongly contributed to PC1 differences between tendon (top row) vs fibrocartilage (bottom row) phenotypes. n=3 independent samples. Data shown as mean+/-SD.

Supplementary Figure 9

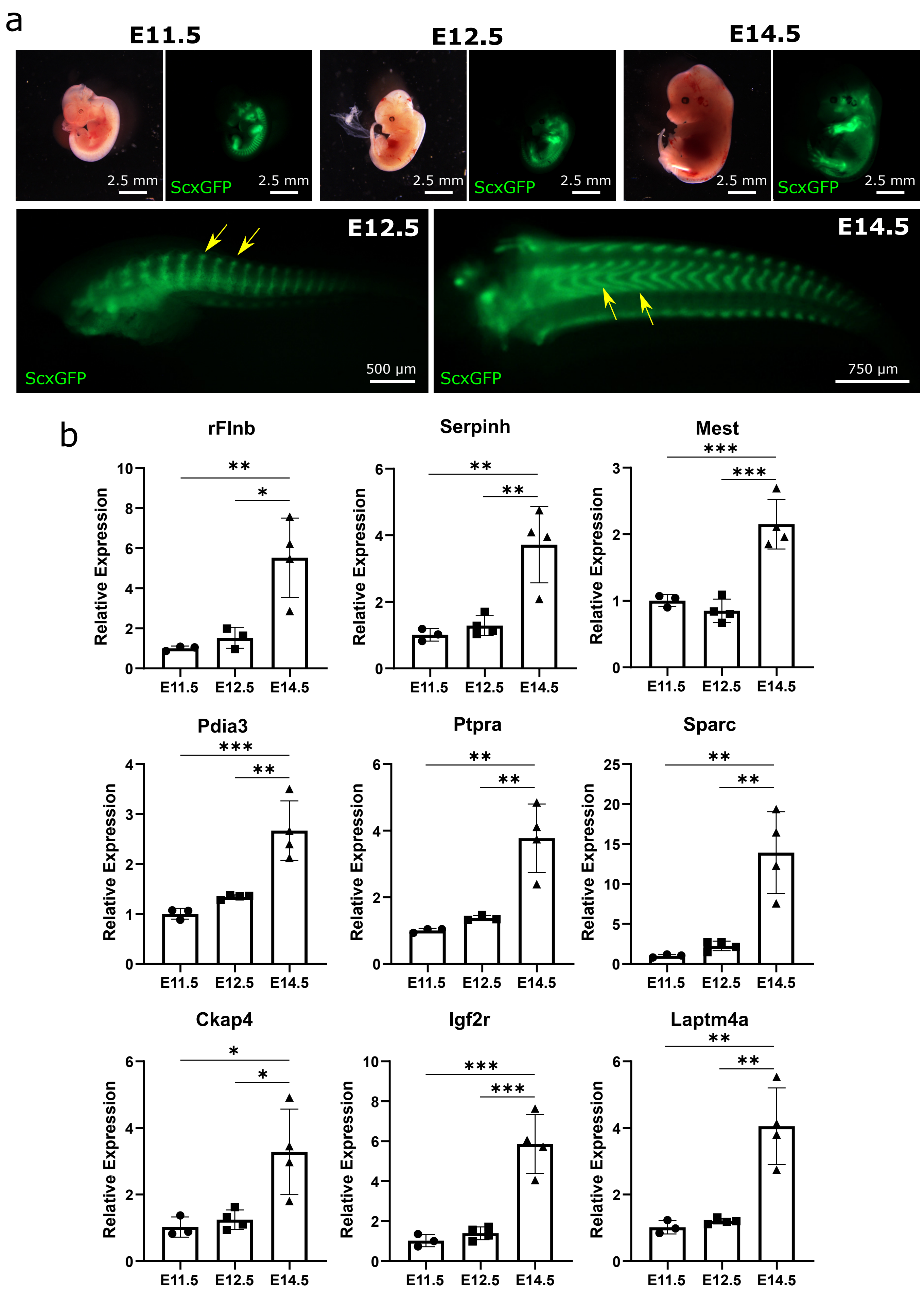

Supplementary Figure 9: In vivo validation of fate markers identified from RNA-seq using embryonic mouse tails. (A) Whole mount bright field and ScxGFP images of embryos collected at E11.5, E12.5 and E14.5. White arrows indicate ScxGFP+ syndetome at E12.5 and elongating tendon elements at E14.5. (B) Real time qPCR quantification of fate-independent markers identified from RNA-seq (n=3 E11.5 embryos and n=4 E12.5 and E14.5 embryos, one way ANOVA with Tukey's posthoc tests). Data shown as mean+/-SD. *p<0.05 **p<0.01 ***p<0.001. Source data provided in source data file

Supplementary Figure 10

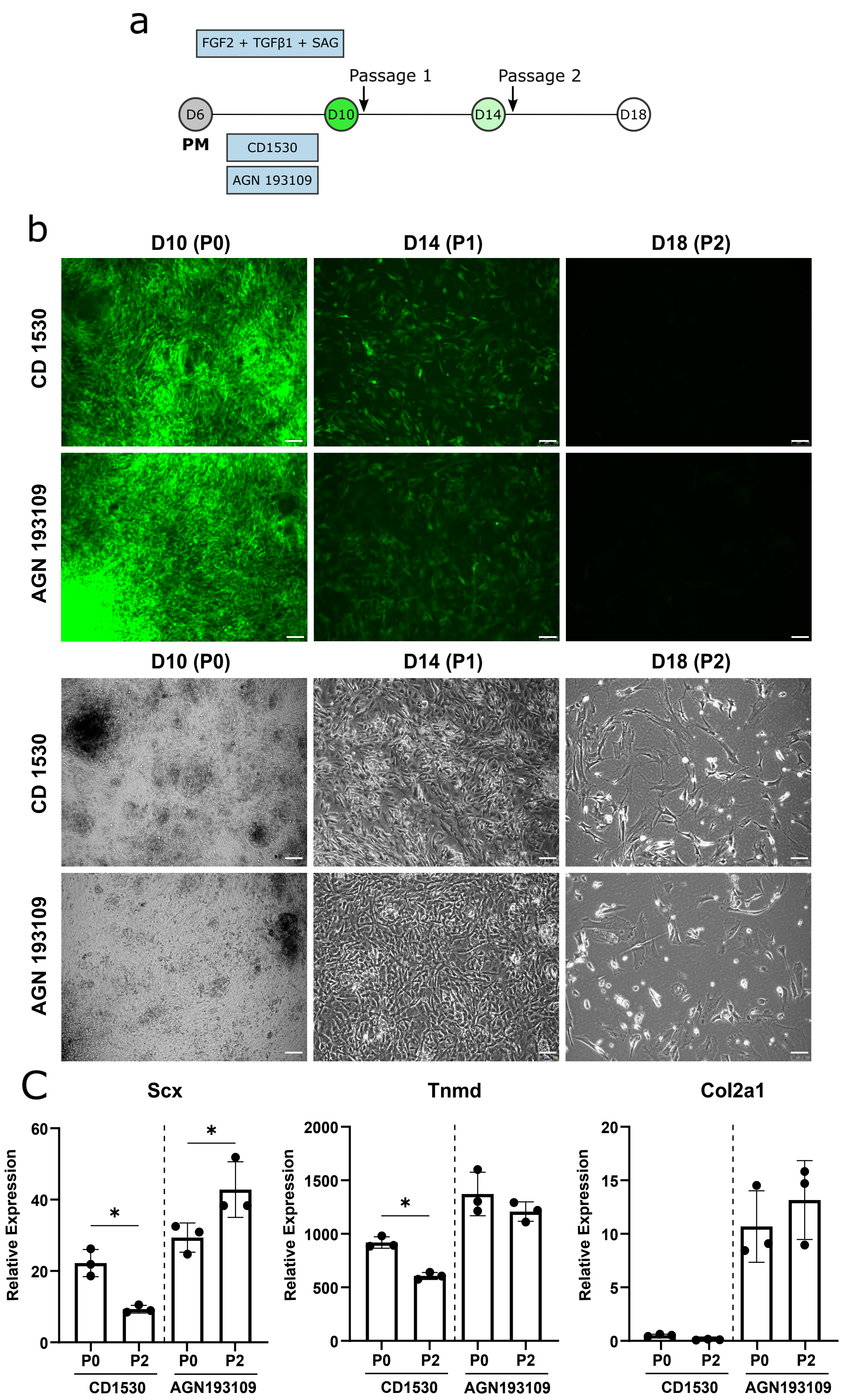

Supplementary Figure 10: Extended 2D culture and passaging results in loss of ScxGFP and proliferative capacity. (A) Schematic of experimental design. Top row indicates common media components. Bottom row indicates media component variables. Medias indicated were maintained throughout D6-D18. (B) Fluorescent and phase contrast imaging of cells. Scale: 100 um. Experiment was repeated three times. (C) Real time qPCR analysis of tendon (Scx, Tnmd) and cartilage (Col2a1) at passage P0 (D10) and passage P2 (D18) (n=3 independent samples, unpaired two-sided Student's t-tests). Data shown as mean+/-SD. *p<0.05. Source data provided in source data file.

Supplementary Figure 11

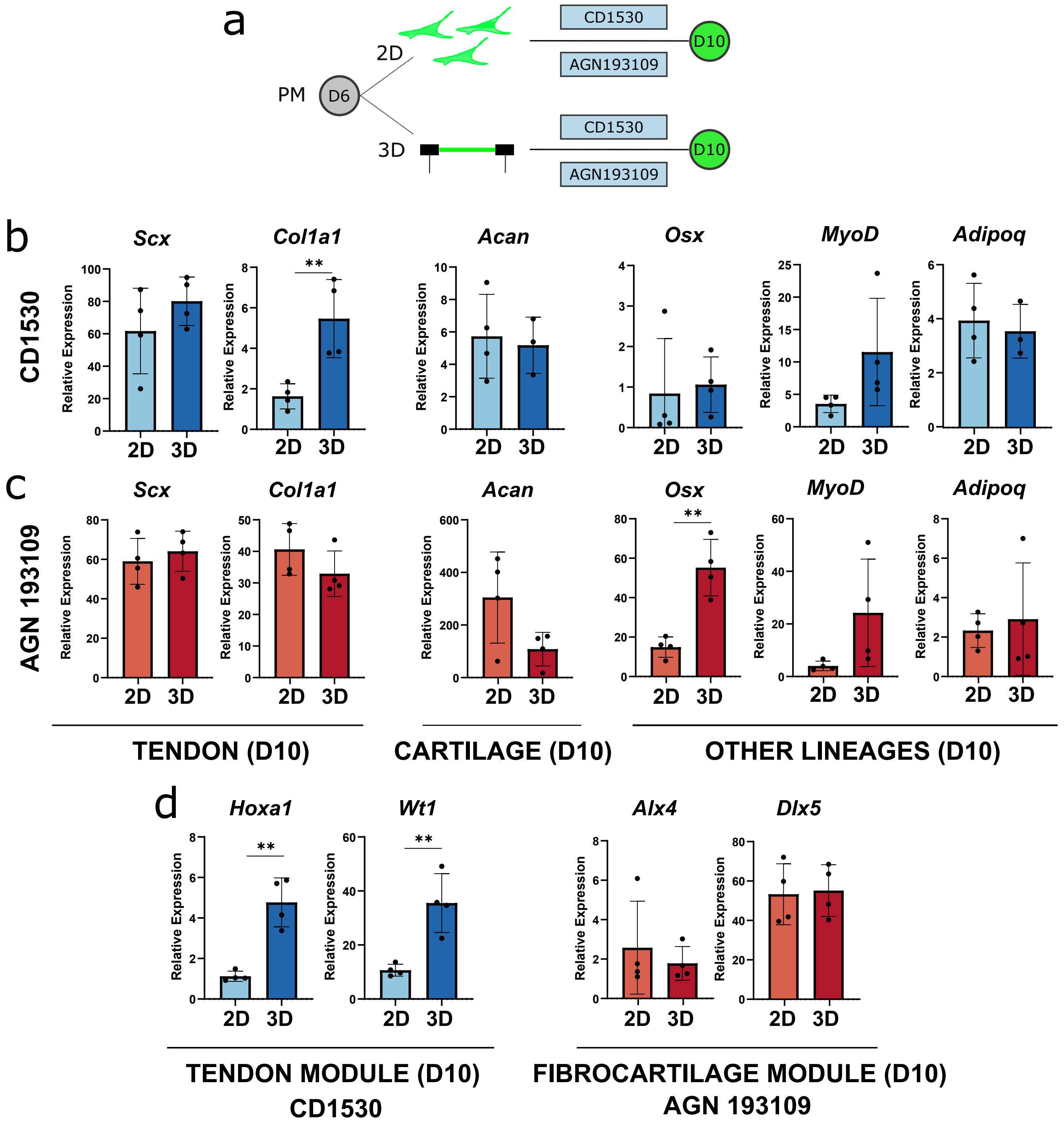

Supplementary Figure 11: Comparison of 2D and 3D culture on tendon and fibrocartilage induction. (A) Schematic of experimental design. 2D condition represents the standard protocol used in prior sections. All media conditions include FGF2, TGFβ1, and SAG from D6-D10. (B-D) Real time qPCR quantification of 2D and 3D cells at D10 for CD1530 and AGN 193109 conditions (n=4 independent samples and gels, unpaired two-sided Student's t-tests). Data shown as mean+/- SD. **p<0.01. Source data provided in source data file.

Supplementary Figure 122

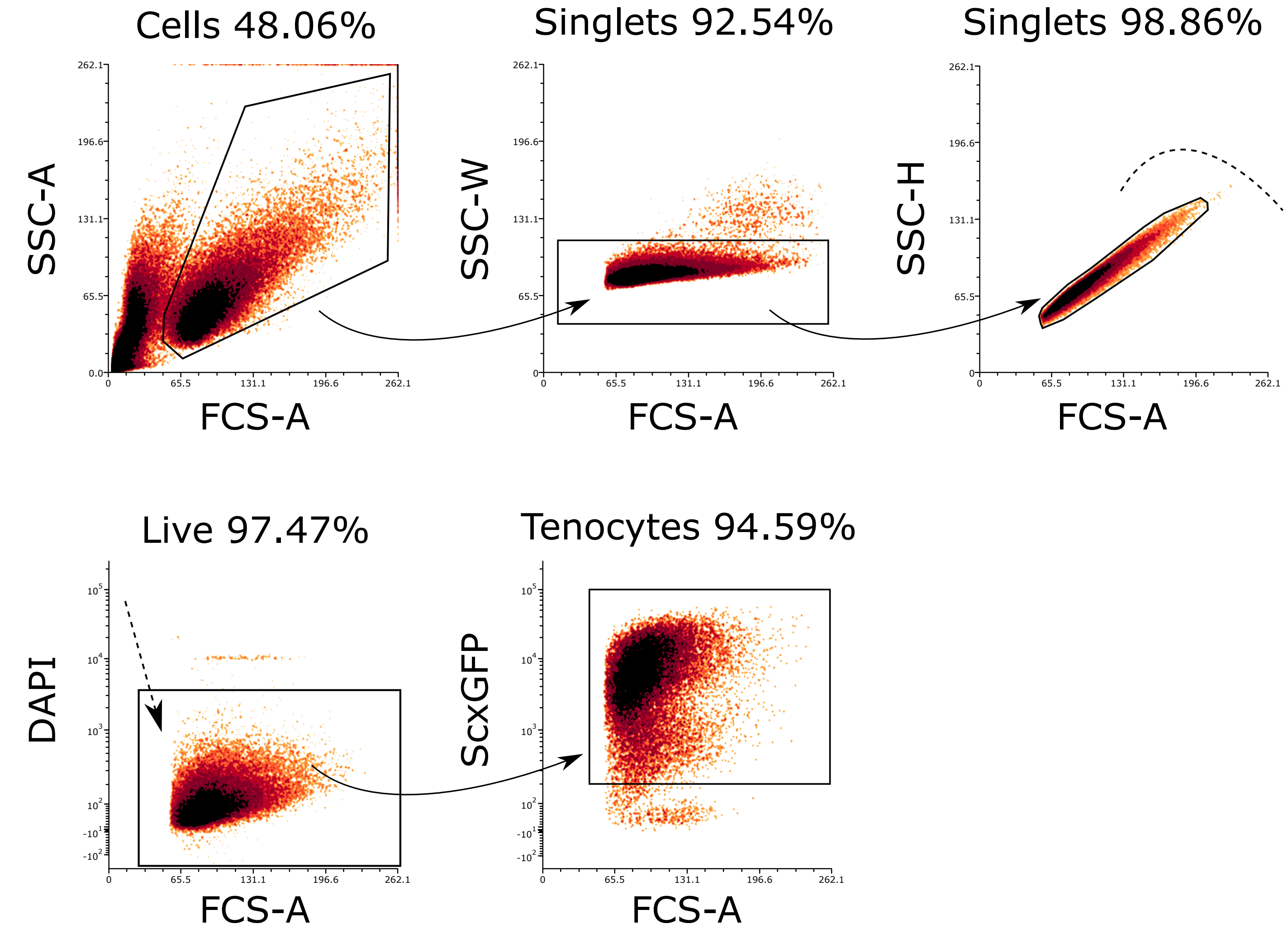

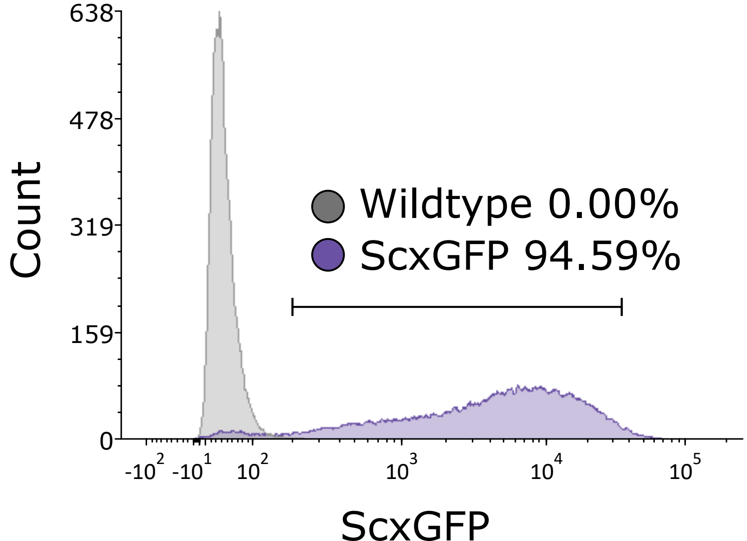

Supplementary Figure 12: Flow cytometry gating strategy for ScxGFP. CCells areggated for live singlets and ScxGFP expression determined based on wild type ScxGFP-negative mESCs cultured in parallel. Gating is used in fig 1b, 1e, 1f, 3d, 3h and supplementary fig 2b.

Supplementary Table 1: Primers used for real time qPCR analyses.

Gene	Fwd Primer	Rev Primer
Gapdh	CCATGACAACTTTGGCATTG	CCTGCTTCACCACCTTCTTG
Scx	AGAAAGTTGAGCAAAGACCGTG	TCAGTGGCATCCACCTTCAC
Mkx	CGTGACAACCCGTACCCTAC	TTTGACACCTGCACTAGCGT
Tnmd	GGGCTGTCACATTCTAAATGCAG	TTCTTCTTCTCGCCGTTGCT
Sox9	CGTGCAGCACAAGAAAGACC	GGACCCTGAGATTGCCCAGA
Col2a1	ATCTTGCCGCATCTGTGTGT	GGCCCTAATTTTCCACTGGC
Nanog	CGGTTCATCATGGTACAGTC	CAGGTGTTTGAGGGTAGCTC
Oct-4 (pou5f1)	CTGTAGGGAGGGCTTCGGGCACTT	CTGAGGGCCAGGCAGGAGCACGAG
Sox2	TTGCCTTAAACAAGACCACGAAA	TAGAGCTAGACTCCGGGCGATGA
Col11a1	GGCCAAAGGAGAAACCAGGAAG	GGGCAGAGGCAGTCAGGAGCT
Matn4	TTAGCACCATGACGCACCT	GGACTCCGAAGCTCTGTCC
Acan	TTCACTGTAACCCGTGGACT	TGGTCCTGTCTTCTTTCAGC
Pax1	GCTCAGTACATTTGTTAATTTTGAAGAAC	TTGCCCACCCCAATTCTTT
rFlnb	ACCCCTATAGTCCCGAACCC	GAAGTGCCGCTCAGAGTCAT
Serpinh1	CGCCATGTTCTTTAAGCCACACT	CATGAAGCCACGGTTGTCCA
Mest	GAGTGGTGGGTCCAAGTAGG	ACCACACCGACAGAATCTTGG
Pdia3	TGCCTCAGTGGTGGGTTTTT	TGTTGGTGTGTGCAAATCGG
Ptpra	GAATGACAAGATGCGCACGG	TCTTTCTGCCGGTATCCATCA
Spac	CACCTGGACTACATCGGACC	TCATGGATCTTCTTCACACGCA
Ckap4	GGCTGGTATGTCCATCACGTC	GCTTGCAGGGATTGGACCTTCT
Igf2r	CTTGCCCTCCAGAAACGGAT	TGCTACACCACAGTTTCGCT
Laptm4a	GCCGAAAGCTTTTGTCAGGG	ACAGGTTGACCACCATGTACC
Osx	ATGGCGTCCTCTCTGCTTG	TGAAAGGTCAGCGTATGGCTT
MyoD	AAGACGACTCTCACGGCTTG	GCAGGTCTGGTGAGTCGAAA
Adipoq	GAAGCCGCTTATGTGTATCGC	GAATGGGTACATTGGGAACAGT

Hoxa1	CCCAGACGGCTACTTACCAGA	CATAAGGCGCACTGAAGTTCT
Wt1	AGCACGGTCACTTTCGACG	GTTTGAAGGAATGGTTGGGGAA
Alx4	ACTTGCGTCTCTTACTGCGAG	TAAAAGGCGACGAACCTTCCC
Dlx5	CTGGCCGCTTTACAGAGAAG	CTGGTGACTGTGGCGAGTTA
Yap1	ACCCTCGTTTTGCCATGAAC	TGTGCTGGGATTGATATTCCGTA
Wwtr1	GTGTGCCCAATGCACTGAC	TGACGCATCCTAATCCTCTCTC
Tpv4	AAACCTGCGTATGAAGTTCCAG	CCGTAGTCGAACAAGGAATCCA
Mrtfa	ACGAGGCGGTTACCATCAC	GCAGACAGAGACAGGAGCAC
