# Supplementary material for: Transcriptional profiling of mESC-derived tendon and fibrocartilage cell fate switch
Source: Nat Commun. 2021 Jul 9;12:4208. doi: 10.1038/s41467-021-24535-5 (PMC8270956; doi:10.1038/s41467-021-24535-5)
Supplement: Supplementary file 3 — Description of Additional Supplementary Files [file 41467_2021_24535_MOESM3_ESM.docx]

Description of Additional Supplementary Files

Title: Supplementary Data 1

Description: Differentially expressed genes for each cluster identified from single cell RNA sequencing of E14.5 mouse tail cells. Adjusted p values were calculated based on general linera model and negative binomial distribution fits followed by likelihood ratio testing.

Title: Supplementary Data 2

Description: Differentially expressed genes between Tendon (Cluster 0) and Fibrocartilage (Cluster 1). Adjusted p values were calculated based on general linera model and negative binomial distribution fits followed by likelihood ratio testing.

Title: Supplementary Data 3

Description: Differentially expressed genes for each cluster identified from single cell RNA sequencing of mESC-derived paraxial mesoderm cells. Adjusted p values were calculated based on general linera model and negative binomial distribution fits followed by likelihood ratio testing.

Title: Supplementary Data 4

Description: Genes comprising fate-independent and fate-dependent modules identified from RNA sequencing of tendon and fibrocartilage differentiation trajectories. Adjusted p values were calculated based on general linera model and negative binomial distribution fits followed by likelihood ratio testing.
