# Supplementary material for: Transcriptional profiling of mESC-derived tendon and fibrocartilage cell fate switch
Source: Nat Commun. 2021 Jul 9;12:4208. doi: 10.1038/s41467-021-24535-5 (PMC8270956; doi:10.1038/s41467-021-24535-5)
Supplement: Supplementary file 8 — Reporting Summary [file 41467_2021_24535_MOESM8_ESM.pdf]

nature research

Corresponding author(s): Alice H. Huang

Last updated by author(s): June 13, 2021

Reporting Summary

Nature Research wishes to improve the reproducibility of the work that we publish. This form provides structure for consistency and transparency in reporting. For further information on Nature Research policies, see our Editorial Policies and the Editorial Policy Checklist.

Statistics

For all statistical analyses, confirm that the following items are present in the figure legend, table legend, main text, or Methods section.

n/a	Confirmed	
☐	☒	The exact sample size (*n*) for each experimental group/condition, given as a discrete number and unit of measurement
☐	☒	A statement on whether measurements were taken from distinct samples or whether the same sample was measured repeatedly
☐	☒	The statistical test(s) used AND whether they are one- or two-sided<br>*Only common tests should be described solely by name; describe more complex techniques in the Methods section.*
☐	☒	A description of all covariates tested
☐	☒	A description of any assumptions or corrections, such as tests of normality and adjustment for multiple comparisons
☐	☒	A full description of the statistical parameters including central tendency (e.g. means) or other basic estimates (e.g. regression coefficient) AND variation (e.g. standard deviation) or associated estimates of uncertainty (e.g. confidence intervals)
☐	☒	For null hypothesis testing, the test statistic (e.g. *F*, *t*, *r*) with confidence intervals, effect sizes, degrees of freedom and *P* value noted<br>*Give P values as exact values whenever suitable.*
☒	☐	For Bayesian analysis, information on the choice of priors and Markov chain Monte Carlo settings
☐	☒	For hierarchical and complex designs, identification of the appropriate level for tests and full reporting of outcomes
☐	☒	Estimates of effect sizes (e.g. Cohen's *d*, Pearson's *r*), indicating how they were calculated

Our web collection on statistics for biologists contains articles on many of the points above.

Software and code

Policy information about availability of computer code

Data collection	FACSDiva v8.0.3
Data analysis	FCS Express v7, CellRanger v3.1.0, bcl2fastq v2.20, Seurat v3.0, STAR Aligner v2.7, R v3.6.3, Python 3.7.3, featureCounts v1.6.0, edgeR v3.28.1, SciPy v1.3.0, Matplotlib v3.1.0, enrichR (2016 version), Image J v1.53e, ggplot2 v3.3.0, HiSeq Control Software v3.6.3, Seaborn v0.9.0

For manuscripts utilizing custom algorithms or software that are central to the research but not yet described in published literature, software must be made available to editors and reviewers. We strongly encourage code deposition in a community repository (e.g. GitHub). See the Nature Research guidelines for submitting code & software for further information.

Data

Policy information about availability of data

All manuscripts must include a data availability statement. This statement should provide the following information, where applicable:

- Accession codes, unique identifiers, or web links for publicly available datasets
- A list of figures that have associated raw data
- A description of any restrictions on data availability

scRNA-Seq and RNA-Seq data has been submitted and is available in the GEO repository database (GSE154525, GSE154397).

Field-specific reporting

Life sciences study design

All studies must disclose on these points even when the disclosure is negative.

Sample size	Sample size was determined based on previous data for gene expression in cell cultures (see https://www.ncbi.nlm.nih.gov/pmc/articles/PMC2928043/). For flow cytometry, sample size was calculated based on initial pilot data showing n=4 sufficient to detect 20% difference in means with CV of 10% (power 0.8 and alpha = 5%). For quantification of immunostaining, sample size was determined based on our previous data (see https://elifesciences.org/articles/51779).
Data exclusions	qPCR data was excluded based on outlier testing
Replication	All cell culture experiments were repeated at least three times and at least two mESC lines were used, showing successful replication.
Randomization	From a given experiment, groups were randomly allocated to particular treatments
Blinding	Blinding was only carried out for immunostaining quantifications. For other assays, blinding was not carried out but all analyses were performed at the same time for all groups of a given experiment. For these experiments, blinding was not relevant since all conditions were subjected to the same analyses (for example, flow cytometry gating) and all qPCR analyses was carried out by Core facilities.

Reporting for specific materials, systems and methods

We require information from authors about some types of materials, experimental systems and methods used in many studies. Here, indicate whether each material, system or method listed is relevant to your study. If you are not sure if a list item applies to your research, read the appropriate section before selecting a response.

Materials & experimental systems

n/a	Involved in the study	
☒	☐	Antibodies
☐	☒	Eukaryotic cell lines
☒	☐	Palaeontology and archaeology
☐	☒	Animals and other organisms
☒	☐	Human research participants
☒	☐	Clinical data
☒	☐	Dual use research of concern

Methods

n/a	Involved in the study	
☒	☐	ChIP-seq
☐	☒	Flow cytometry
☒	☐	MRI-based neuroimaging

Eukaryotic cell lines

Policy information about cell lines

Cell line source(s)	mouse embryonic stem cells were derived from ScxGFP mouse blastocysts. ScxGFP-negative lines were also generated from littermates and used for flow gating purposes
Authentication	lines were derived and authenticated by Dr. Kevin Kelley from Mount Sinai's Transgenic Animal Core Facility. ScxGFP+ lines were identified by genotyping with validated primers provided by Dr. Schweitzer who generated the mouse.
Mycoplasma contamination	cell lines were not tested
Commonly misidentified lines (See ICLAC register)	No commonly misidentified lines were used in this study.

Animals and other organisms

Policy information about studies involving animals; ARRIVE guidelines recommended for reporting animal research

Laboratory animals	Mouse embryos (E11.5, E12.5, E14.5) were derived from ScxGFP mice on C57BL6 strain and used in accordance with IACUC approved guidelines. Embryos were staged based on time of conception and both male and female embryos were used.
Wild animals	no wild animals were used in these studies
Field-collected samples	no field collected samples were used in these studies
Ethics oversight	All procedures were carried out under approval by the Institutional Animal Care and Use Committee at the Icahn School of Medicine at Mount Sinai

Note that full information on the approval of the study protocol must also be provided in the manuscript.

Flow Cytometry

Plots

Confirm that:

- [x] The axis labels state the marker and fluorochrome used (e.g. CD4-FITC).
- [x] The axis scales are clearly visible. Include numbers along axes only for bottom left plot of group (a 'group' is an analysis of identical markers).
- [x] All plots are contour plots with outliers or pseudocolor plots.
- [x] A numerical value for number of cells or percentage (with statistics) is provided.

Methodology

Sample preparation	Cells were trypsinized and resuspended in 2% FBS in PBS and stained with DAPI prior to flow
Instrument	LSRIIA
Software	FACSDiva was used for data acquisition and data analyzed by FCS Express 7
Cell population abundance	ScxGFP cells were highly abundant, representing nearly 20% of the base media condition and over 70% of the positive treatment groups.
Gating strategy	FSC/SSC was gated to include the entire population. ScxGFP gating was set based on a ScxGFP-negative mESC line (derived from littermates) that underwent the same experimental condition.

- [x] Tick this box to confirm that a figure exemplifying the gating strategy is provided in the Supplementary Information.
